# Data Augmentation of Automotive LIDAR Point Clouds under Adverse Weather Situations

**DOI:** 10.3390/s21134503

**Published:** 2021-06-30

**Authors:** Jose Roberto Vargas Rivero, Thiemo Gerbich, Boris Buschardt, Jia Chen

**Affiliations:** 1 Audi AG, Auto-Union-Str., D-85057 Ingolstadt, Germany; jose-roberto.vargas-rivero@audi.de (J.R.V.R.); thiemo.gerbich@audi.de (T.G.); boris.buschardt@audi.de (B.B.); 2 Electrical and Computer Engineering, Technical University of Munich, Theresienstr. 90, D-80333 München, Germany; 3 Institute for Advanced Study, Technical University of Munich, Lichtenbergstraße 2 a, D-85748 Garching, Germany

**Keywords:** LIDAR, point cloud, spray water, ADAS, AV, data augmentation, classification, synthetic data, sensor model

## Abstract

In contrast to previous works on data augmentation using LIDAR (Light Detection and Ranging), which mostly consider point clouds under good weather conditions, this paper uses point clouds which are affected by spray. Spray water can be a cause of phantom braking and understanding how to handle the extra detections caused by it is an important step in the development of ADAS (Advanced Driver Assistance Systems)/AV (Autonomous Vehicles) functions. The extra detections caused by spray cannot be safely removed without considering cases in which real solid objects may be present in the same region in which the detections caused by spray take place. As collecting real examples would be extremely difficult, the use of synthetic data is proposed. Real scenes are reconstructed virtually with an added extra object in the spray region, in a way that the detections caused by this obstacle match the characteristics a real object in the same position would have regarding intensity, echo number and occlusion. The detections generated by the obstacle are then used to augment the real data, obtaining, after occlusion effects are added, a good approximation of the desired training data. This data is used to train a classifier achieving an average F-Score of 92. The performance of the classifier is analyzed in detail based on the characteristics of the synthetic object: size, position, reflection, duration. The proposed method can be easily expanded to different kinds of obstacles and classifier types.

## 1. Introduction

The high degree of reliability required by newer ADAS and AV functions makes the combination of different sensors necessary [1,2,3]. Among those sensors, radar, cameras, ultrasound and GPS (global positioning system) are common. Recently, LIDAR sensors have also been used mostly due to their high resolution, ability to work under low light conditions and direct distance measurement [4,5]. However, the high resolution obtained by using infrared light comes at the cost of a reduced performance when dirt [6], rain, fog [7], snow [8], exhaust gases [9] and spray water [10] are present. Besides reducing range, these phenomena cause extra detections which can have negative effects like phantom braking. Mechanisms to filter out those extra detections are difficult to implement, as there is a risk of removing detections caused by real objects. 

**Figure 1:**
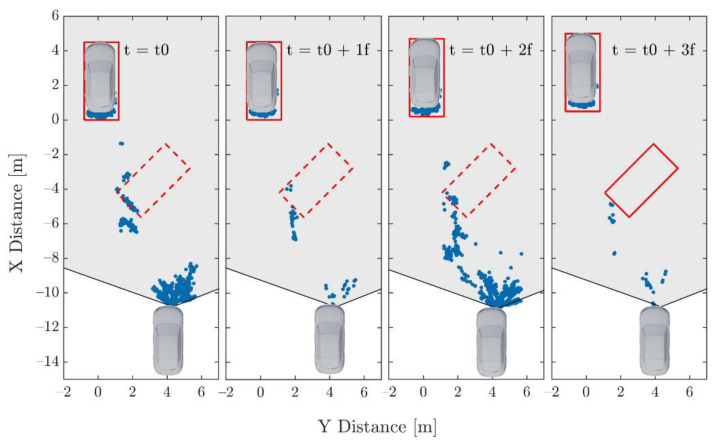
Bird’s eye view of a sequence in which a ghost object is generated due to spray water. A wrong object hypothesis created at the beginning (dashed line) is not rejected in subsequent frames; as a consequence, an extra object is generated (solid line). The field of view of the sensor is shown in light grey (based on [10]).

In this context, this paper focuses on correctly classifying solid objects in point clouds affected by spray water. Spray water is especially problematic because it can generate point clouds with characteristics that highly resemble those of solid objects. Figure 1 shows a real example in which the spray cloud generates detections with a shape close to the ‘L’ shape of a vehicle [10]. True positives are needed to train a classifier to remove this kind of false positive. This corresponds to cases in which real solid objects are present simultaneously with spray, as caused, for example, by objects falling off of a vehicle. As the amount of spray shown in Figure 1 is usually generated at highway speeds, collecting this kind of data is extremely dangerous even if done at a proving ground. Even using a high number of data collecting vehicles, the number of examples obtained may be too low for training.

### State of the Art

Regarding data augmentation, there have been multiple approaches going from transformations of a real training set [11,12,13,14,15,16], to combinations of real and synthetic data [17,18,19,20], to purely synthetic data [21,22,23] and domain adaptation techniques [24,25,26]. In [12], the point clouds of previously labeled objects are added by concatenation at different positions into the training data in order to improve the training of the network. Possible collisions between added objects are automatically removed. In [15], an adversarial strategy is used in order to simultaneously train an ‘augmentor,’ which is a neural network specialized in creating augmented data which causes a reduction in the performance of a classifier. When trained simultaneously, the classifier learns features that are independent of the possible modifications caused by the augmentor. As there is a broad range of operations that can be applied for augmentation, in [16] a search method is proposed in order to find the optimal augmentation policy, understood as a series of augmentation operations with their respective parameters. 

Similar to our method in [17], real scans are used for the background which corresponds to portions of streets in which the movable objects and pedestrians are removed. Synthetic movable objects and pedestrians are then placed with a number, position and pose based on probabilities extracted from labeled real datasets. A sensor model is then used to generate the final point clouds. This permits the generation of high amounts of annotated 3D data for training. A similar approach is used in [18]. In this case, a synthetic object is added on top of the roof of vehicles in real point clouds. This synthetic object is modeled in order to attack the point cloud-based classification algorithm, reducing its accuracy and hence identifying possible vulnerabilities. 

Unique to our approach is the use of real point clouds affected by spray water. The collection of this data and the setup used are explained in Section 2. The ROI (region of interest) in which the synthetic object is generated is based on previous results regarding the spatial distribution of spray and the possible actions that can be taken to protect the passengers. 

As the synthetic object needs to match the characteristics of a real object placed in the ROI, spray itself has to be simulated. As done in previous works, the scene is rendered. In our case, a physically based render is used to simulate the LIDAR sensor [27] with material properties based on real reflection values. Spray and rain are simulated using particle systems which generate detections with a spatial distribution similar to those caused by real spray [10]. After rendering, the detections caused by the obstacle are extracted and concatenated into the real point clouds. Finally, the effect of occlusion and noise are added. 

With the obtained point clouds, a two-layer feedforward neural network is trained. The results are presented in Section 3. The classifier is trained to identify detections caused by the added obstacle. The method uses a bird’s eye view of the 3D point cloud in which detections are assigned to bins in a 2D histogram similar to what is done in [28,29] for the extraction of vehicle bounding boxes. The obtained histogram(s) is then convoluted with objects having the dimensions and orientation corresponding to the features that need to be extracted. The use of the bird’s eye view with fixed histogram sizes and a simple network for classification allows to keep the required time budget. Classification based on detections instead of a binary classification of the whole scene has as advantage that the decision to activate any safety mechanism can be made based on the extracted obstacle: dimensions, position, rotation and/or reflection and the characteristics of the ego vehicle, such as its ground clearance, for example. The results are discussed in Section 4.

## 2. Experiment

### 2.1. Region of Insterest

**Figure 2:**
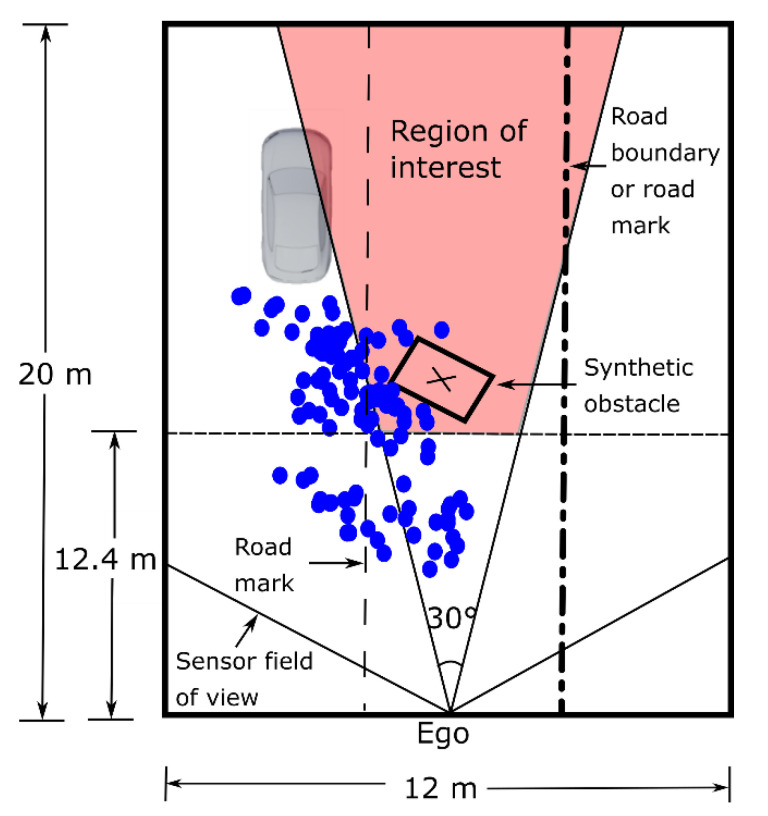
Region of interest. The size is based on the region in which spray is usually detected [10] for the LIDAR and speeds used. Before 12.4 m, even if a solid object is detected no meaningful action can take place. The 30° are based on the curve radii found in European roads. The ‘x’ marks the center of the added synthetic object.

**Figure A1:**
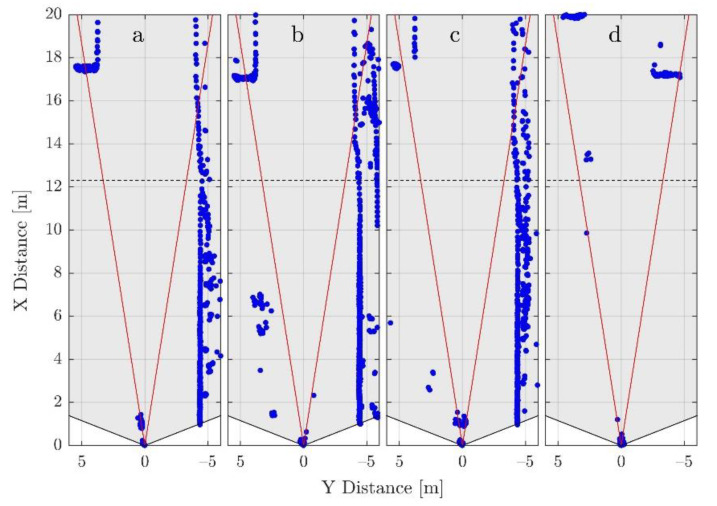
Examples of the point clouds generated by different road boundaries (**a**–**c**) and vehicles in other lanes (**d**). The ROI is limited in ‘y’ by the read lines and in ‘x’ by the dotted line.

The region at which spray detections are usually seen, based on our measurements on the highway and considering that the spray is generated by one leading vehicle (LV), corresponds approximately to 6 m to each side of the ego vehicle and up to 20 m in front of it [10]. These measurements were taken with an average ego speed of 89 km/h. Under these circumstances, if a solid object is present in the spray cloud, it is not possible to correct the vehicle trajectory in time. An emergency stop would also not be enough to stop the vehicle before hitting the possible object and would be quite dangerous. There are, however, corrective measures that can be taken. The actuator of the seatbelt pretensioner can be activated as fast as 100 ms to 300 ms [30]. There are also numerical studies regarding the rotation of the seats, in which rotations up to 90° under 200 ms are considered possible and useful in order to protect the passengers [31]. Additionally, a slight brake can be applied. Taking 200 ms as the average for the actuator and adding 300 ms for the detection and classification of the object yields a total of 500 ms. Based on the previously mentioned average speed of our spray measurements, this corresponds to a distance of 12.36 m. Additionally, in order to take curved roads into account and the possibility that a falling object moves from an adjacent lane to the ego lane, we used a cone-shaped ROI. The angle is based on the value of the vehicle exit angle for European roads, which corresponds to the angle between the road edge line and the vehicle straight trajectory [32]. An angle of 30°, which should cover most of the left and right curves found in European highways, was chosen. The final ROI is shown in Figure 2. During the measurements the ego vehicle occupied either the second or third lane while the LV occupied the first or second lane, respectively. For this reason, to the right there could be either a road boundary or another lane. This makes the classification more challenging, as can be seen in Figure A1 in Appendix A.

### 2.2. Measurement

**Figure 3:**
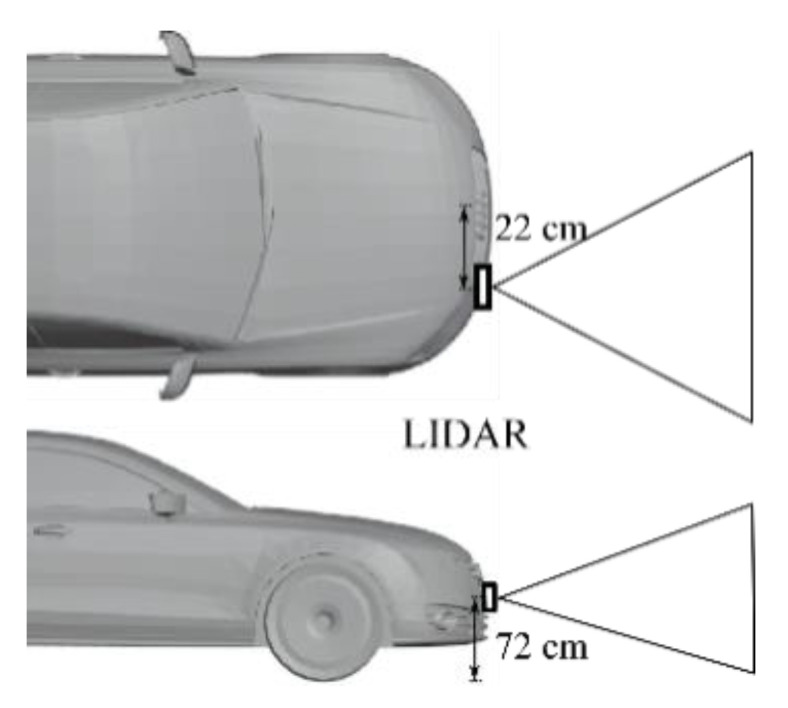
Placement of the LIDAR sensor in the ego vehicle (based on [10]).

An automotive LIDAR sensor on the position indicated in Figure 3 was used for data collection. The measurement was taken on a highway during the dates indicated in Table 1. Those days were rainy days. The rainfall during the measurement as reported by nearby weather stations is also indicated [10].

A total of 13.162 frames were collected. In each measurement there is a LV, which generates spray, located in the lane to the left of the ego vehicle. The position of the LV was tracked using the internal tracker of the sensor and is reported in Figure A3. The ego speed had a value of (89 ±4) km/h. 

### 2.3. Simulation 

**Figure 4:**
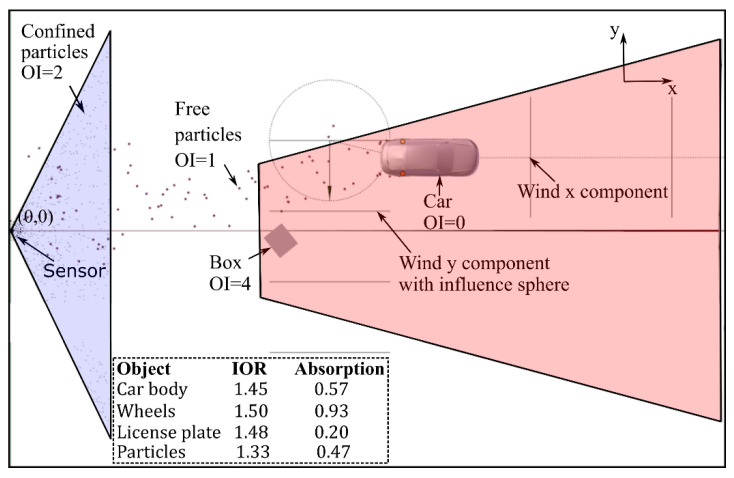
Simulation setup. The object index (OI) is a parameter used by the render to create a separate mask for the object to add composition effects [34] and makes it is possible to identify which object caused which detections. Notice the correspondence with Figure 2. The ROI is marked in red. The region in violet corresponds to a volume in which confined particles are uniformly distributed to simulate the detections caused by rain. The index of refraction (IOR) and absorption used for the material of each object is shown in the dashed line region [10].

The simulation setup used is presented in Figure 4. This setup is based on the model introduced in [10]. The original simulation setup was based on the following assumptions:

- Simulating real water drops as well as the exact forces that act upon them cannot be done in real time. However, the number of detections that those drops cause is usually much smaller as a high concentration of drops is needed to cause a reflection strong enough to cause a detection. Therefore, a more efficient approach is to use particles to directly simulate detections instead of single drops.
- The solver used by Blender [33] to calculate the trajectories of the particles is stable in the required parameter range.
- The LIDAR sensor itself can be simulated in the same way as a camera image is rendered by adapting the calculation done inside the material shader. The calculation is changed from the default multiplication of intensities to an addition of ray distances. One color channel is left unchanged; as a result, each camera pixel contains the information about the distance that the ray traveled from the light source and its corresponding intensity. The light source is placed next to the camera and both camera and light source are configured based on the resolution and field of view of the sensor.
- If physically based values are used for the materials, as shown inside the dashed line region of Figure 4, the calculated intensities per pixel should be proportional to the ones obtained using the real sensor.

These assumptions were validated by comparing the trajectories of the particles generated by Blender with those generated by a fourth order Runge-Kutta solver for the required parameter range. Additionally, the correlation of the spatial distribution of the real and simulated point clouds was calculated using the Pearson correlation coefficient obtaining values going from 0.5 to 0.74. The rendering time per frame using an Intel CORE i7-8850H CPU varies from 40 ms (8 particles per wheel per frame) up to 260 ms (50 particles per wheel per frame) [10]. 

In this setup, the camera was configured on the basis of the resolution of the sensor but, instead of a one-to-one relation, a three-to-one relation was used. The extra pixels were used to simulate multiple echoes: secondary and tertiary reflections coming from the same direction. Additionally, the confined particles inside the violet volume were unique to this simulation. These detections were needed in order to make the echo values in simulation and in reality coincide. The number of free and confined particles used is presented in Table 2. 

A box was used as an obstacle. Any other obstacle shape could be used, but we decided to use a box for simplicity in the description of its shape. 

The position of the LV in the simulation was set equal to the real position for the corresponding frame. This means every real frame has an equivalent synthetic frame. The number of free particles in the simulation was based on the number of detections caused by spray in reality [10]. These particles are emitted from the wheels, and their size and the forces that act upon them (i.e., wind x and y components) are calibrated on the basis of physical values and the spatial distribution of spray in real measurements [10]. For this specific simulation, the effect of the wind in y, which originally is simulated using two forces, was reduced to only one and the value of this force increased to direct the spray towards the ROI.

The confined particles were distributed uniformly over the violet volume in Figure 4 but were emitted randomly with a lifetime of one frame. This generates a distribution of detections similar to the one observed in real rain measurements using the same sensor [8,10].

In order to calibrate the intensity of the light source, the accumulated histogram of the real and synthetic echo pulse width (EPW, proportional to the intensity of the reflection [8]) values were compared. This was done before adding the box to the simulation. The intensity of the lamp in the simulation was adjusted to generate a similar intensity range as in the measurements. 

**Figure 5:**
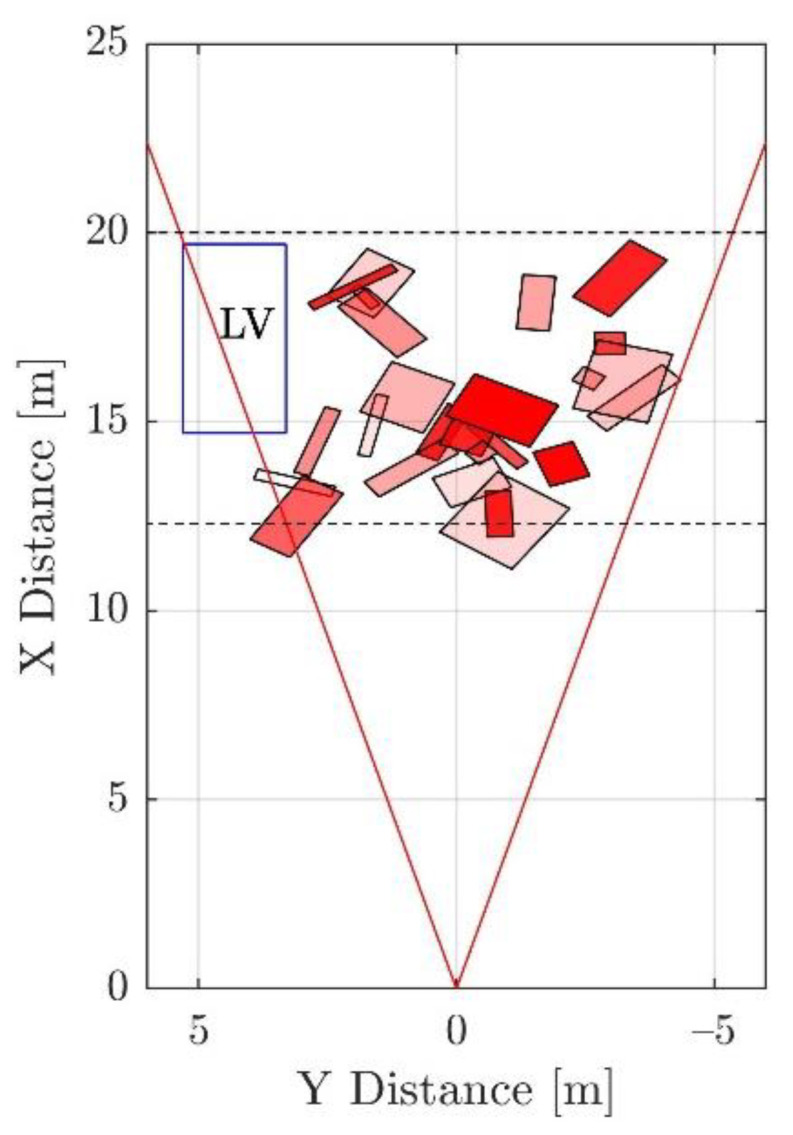
Examples of the generated synthetic obstacles accumulated over multiple frames. The red and dotted lines mark the ROI. The transparency of the box is proportional to its reflection value. The blue box marked ‘LV’ corresponds to the position of the leading vehicle and collisions with it are automatically removed.

After calibration of the light source was completed, the box was added to the simulation. The position of the center of the box is distributed uniformly over the whole ROI. It was verified that there was no collision between the box and the LV. Possible collisions with the point clouds generated by other vehicles or by the road boundary in the real data were dealt with afterwards. The width, length and height of the box were sampled from a uniform distribution going from 20 cm to 2 m. The z position of the box was adjusted to be half its height to ensure that it remained on the street surface. The rotation was uniformly distributed between 0° and 90° as is the reflectivity, which goes from 0 to 1. Its duration, which corresponds to the number of frames in which it remains in the same position, was varied between 1, 2 and 3. An equal number of examples for each duration was used. Figure 5 presents the results obtained by accumulating the boxes generated over multiple frames keeping the LV position constant. The transparency in the boxes in the figure is proportional to their reflection value.

**Figure 6:**
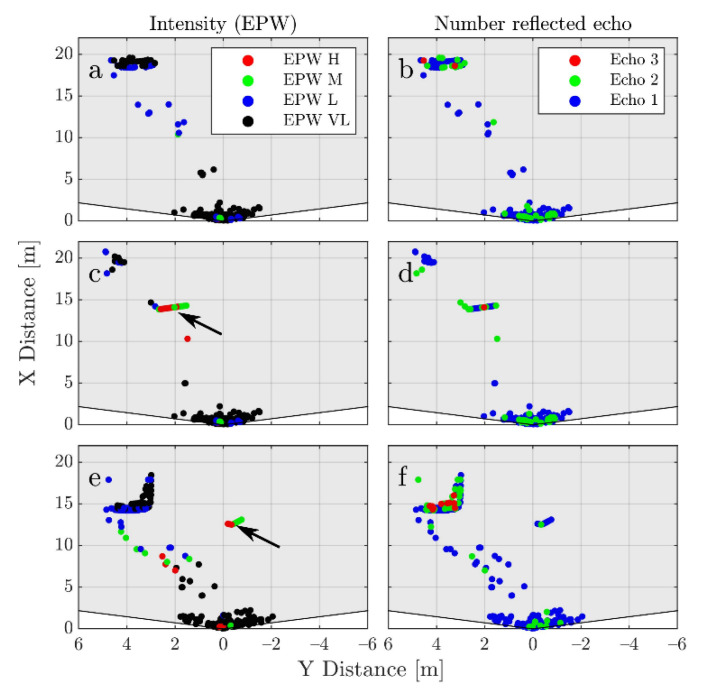
Examples of the generated synthetic frames. On the left side the detections are colored based on the EPW value and on the right are based on the echo number. In (**a**,**b**) there is no added obstacle. In (**c**,**d**) the effect of occlusion caused by the box is shown. In (**e**,**f**) a box that does not cause occlusion is added. The arrows in (**c**,**e**) show the position of the obstacle.

**Figure 7:**
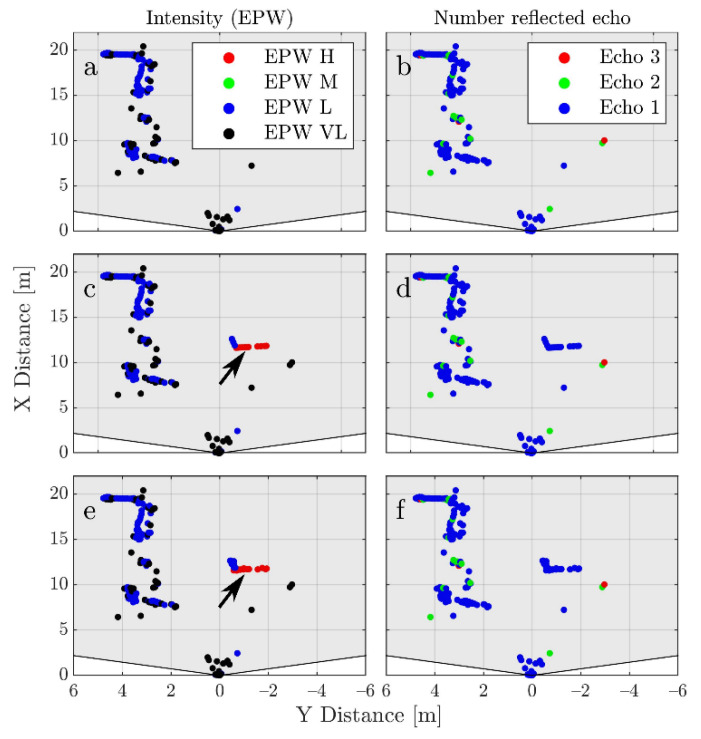
Examples of a real frame before (**a**,**b**), after augmentation (**c**,**d**) and after adding noise (**e**,**f**). On the left side the detections are colored based on the EPW value and on the right based on the echo number. The frame in (**a**,**b**) is the corresponding real frame to the synthetic one shown in Figure 6a,b. The arrows mark the position of the box after augmentation. Notice how the intensity distribution on the box makes physical sense due to the way in which the synthetic data was generated. The echo number is also plausible. The effect of adding noise extracted from real measurements (**e**,**f**) also increases the similarity.

Figure 6 shows the results obtained after rendering. The left side of the figure uses colors for the detections based on their EPW, while the right side uses different colors to identify the corresponding echo number. Three different frames are shown. In Figure 6a,b the box is not added. A direct comparison can be made with Figure 7a,b which corresponds to the real equivalent (same position of LV). As can be noticed, there are similarities in the distributions of the EPWs (before training the classifier the histogram of the EPW values of the detections caused by the synthetic object is adjusted to resemble the one obtained by the real reflections on the rear of the LV (see Figure A4). This is done in order to avoid biasing the classifier towards this difference. The histograms are also trimmed to have the same maximum value.) and echo values. The region close to the sensor (<5 m) contains detections caused mostly by rain, with very low intensity and an echo number of one or two. The detections caused by spray tend to have a very low to low EPW. Mostly first and second echoes are seen with very few third echoes. Something similar happens with the vehicle itself, which is a reason why removing detections caused by spray is difficult. The intensities and echoes are in similar ranges, and only the shape allows for a clear differentiation. 

Figure 6c,d shows a frame in which the box is added, causing occlusion to a portion of the vehicle (the box height goes from 20 cm to 2 m which allows for the occlusion of multiple layers). Notice how the distribution of the intensities in the box makes physical sense with the size perpendicular to the sensor having a higher intensity. The echo number also coincides to what would be expected. Finally, in Figure 6e,f an example is given in which the box does not occlude the vehicle. This occlusion, which happens naturally in the simulation, has to be artificially added when the real data is augmented.

After the synthetic data is generated, the object with OI 4 is extracted and concatenated to the corresponding frame of the measured point clouds. Additionally, Gaussian noise is added to the detections caused by the box. This noise has a standard deviation of 5 cm and mean of zero. The value of the standard deviation is extracted from real reflections caused by the rear of the LV. The effect of adding the noise is shown in Figure 7e,f.

**Figure 8:**
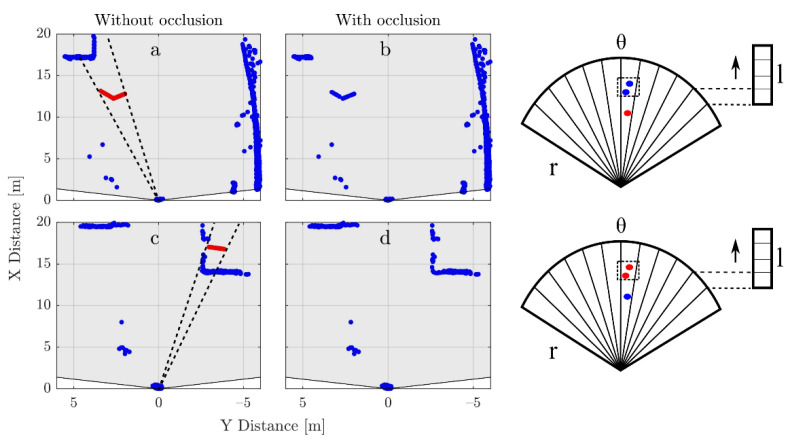
Examples of cases in which occlusion has to be calculated after adding the synthetic object. In (**a**) the detections caused by the LV in the same angular region and layer as the obstacle (dotted line) should be removed. Equivalently in (**c**), the obstacle itself should be removed as its random position happens to be inside another vehicle. The (**b**,**d**), respectively. The angular bin size needs to be selected to avoid strong undersampling of the point cloud if the value is too high. However, if it is too small, the desired occlusion effect does not take place. In our case, a value slightly higher than the resolution of the sensor was used. The occlusion is calculated before the Gaussian noise is added to the synthetic detections.

As previously explained, the cases in which the box intercepts with the LV are automatically removed. It is, however, possible that the box intersects with the road boundary or with vehicles in the adjacent lane as shown in Figure 8c. Additionally, the presence of the box would occlude objects further in the light path as shown in Figure 8a. In order to tackle these two issues, an algorithm based on the radial histogram of the detections per layer is used (Figure 8 right, radial histograms). 

The algorithm works as follow:

1. For each frame, the radial histogram per layer is calculated.
2. The bins that contain detections caused by the box are extracted.
3. The detections inside the bins are organized in ascending order based on their radial distance from the sensor.
4. The first detection is used and the rest are removed. The detections in the region from 0 to 5 m are not taken into consideration as their effect was already included when generating the synthetic data.

Notice that the examples shown in Figure 8a,c and in Figure 6c,d correspond to cases in which all layers are occluded. This is done to clearly identify the effect but would only happen in rare cases in which the synthetic object’s height approaches the upper limit of its range (2 m).

## 3. Results

As a classifier, a feedforward neural network was used with two layers, one hidden layer with 10 neurons and one output layer. As a training function, scaled conjugate gradient backpropagation was used. Cross entropy was used to evaluate performance. 

As output classes: No Box (NB) and Box (B) were used. The NB class included the detections caused by spray, other vehicles, the road boundary and raindrops. The B class corresponded to the detections caused by the synthetic obstacle. In order to compensate for the class imbalance, an error weighting of 20% was used for the NB class and of 100% for the B class.

For training, the region from −6 m to 6 m in ‘y’ and 0 to 20 m in ‘x’ was divided into histograms with different bin sizes. The following bin sizes were used on the basis of the features they should help to identify:

- Small features: 10 cm in ‘x’ by 10 cm in ‘y’.
- Vehicle rear: 20 cm in ‘x’ by 2 m in ‘y’.
- Vehicle side: 4 m in ‘x’ by 10 cm in ‘y’.
- Middle size features: 50 cm in ‘x’ by 50 cm in ‘y’.
- Radial: 1°.

The following classification parameters were used (per detection). The number of counts correspond to those of the bin containing the detection for the corresponding histogram:

1. X position.
2. Y position.
3. Echo number.
4. Layer number.
5. EPW value.
6. Number of counts (radial).
7. Number of counts (small features).
8. Number of counts (vehicle rear).
9. Number of counts (vehicle side).
10. Number of counts (middle size feature).
11. Absolute value of the subtraction of parameter six of the current frame from parameter six of previous frame.
12. Convolution with horizontal matrix (Appendix B) (small features).
13. Convolution with corner matrix^1^ (small features).
14. Convolution with corner matrix^1^ rotated 90° (small features).
15. Convolution with impulse matrix^1^ (small features).

Table 3 presents the classification results using the F-Score as metric. The results are divided between ‘All durations,’ which corresponds to the classification results for durations of one, two and three frames, and ‘Duration 3f’ which contains only the examples in which the object remains in the same position for three frames. The results for durations of one and two frames were very similar and hence are not shown. An F-Score for the B class of 92.13 and 92.26 was obtained, respectively. This indicates, as expected, that the classification improves the longer the box remains in the same position and that adding more parameters like parameter 11 could further improve the results.

**Figure 9:**
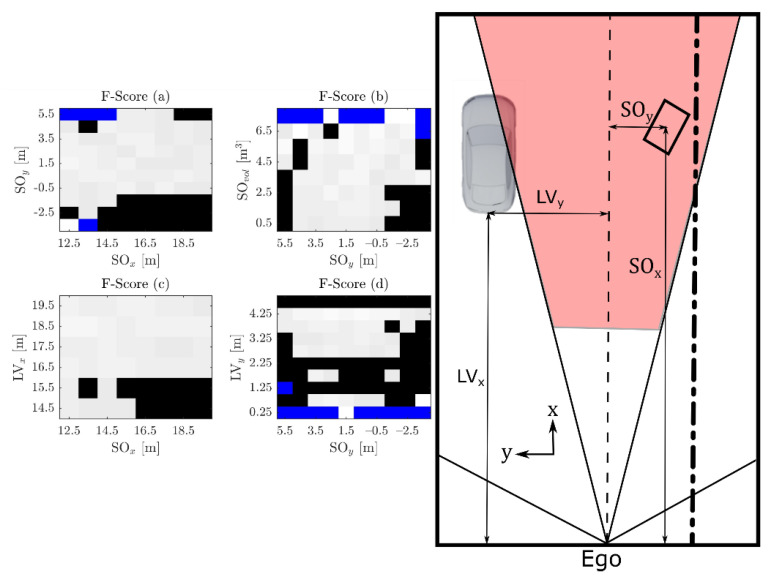
Classification results are analyzed in detail by dividing the available data into small portions. Regions where the F-Score is lower than 92% are shown in black. Blue regions are empty (the combination of parameters currently does not contain any example). On the right, the meaning of each of the parameters is shown. $SO_{vol}$ corresponds to the volume of the synthetic object, $SO_{x}$ and $SO_{y}$ to its coordinates. $LV_{x}$ and $LV_{y}$ correspond to the coordinates of the leading vehicle. In both cases, the position is calculated with respect to the position of the LIDAR sensor on the Ego vehicle. The difficulty of the classification task increases when the synthetic object is located close to the road boundary (**a**,**b**) or when is close to the LV and has a small size (**b**), additionally when the synthetic object is farther away in x-direction as the LV (**c**) or when the LV moves to the Ego lane (**d**).

**Figure A2:**
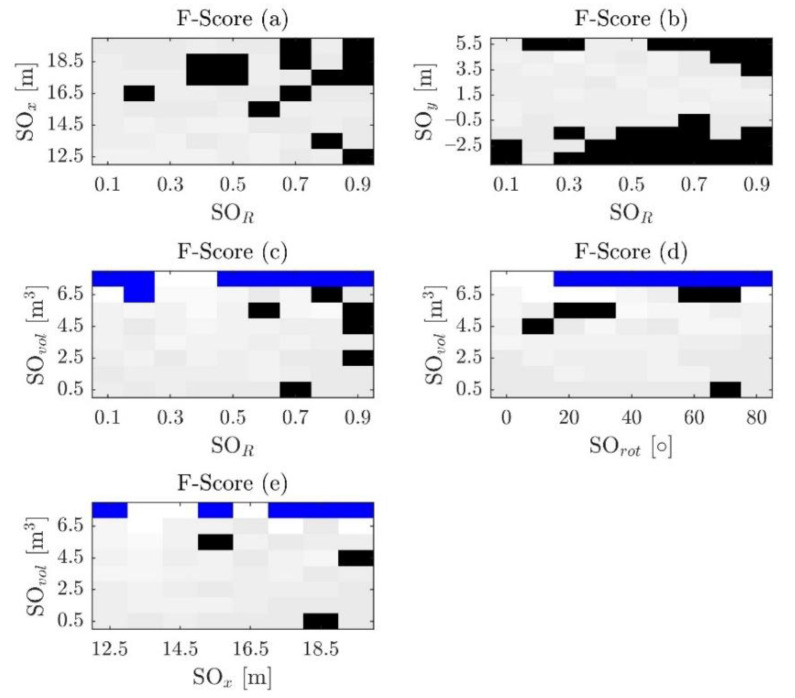
Continuation of Figure 9, including the dependencies for other parameters. In black regions with a F-Score lower than 92% are shown, and blue regions that are empty are shown (the combination of parameters currently does not contain any example). Besides the already explained parameters, $SO_{R}$ corresponds to the reflection of the synthetic object and $SO_{rot}$ to its rotation in degrees. In (**a**–**c**) high reflection values for the synthetic object seem to reduce the F-Score. A high reflection generates a high EPW value, high EPW values are more likely on the LV (Figure A4) making the classification harder. The rotation (**d**) and volume (**c**–**e**) do not seem to have a strong influence by themselves.

**Figure A3:**
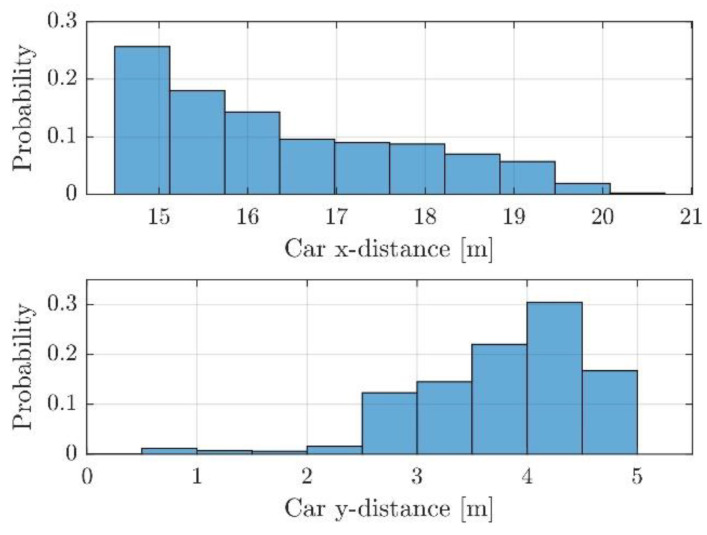
Probability distribution for the position of the LV with respect to the ego vehicle. The reference point in the LV is the middle of the lower side of its bounding box (considering bird’s eye view).

**Figure A4:**
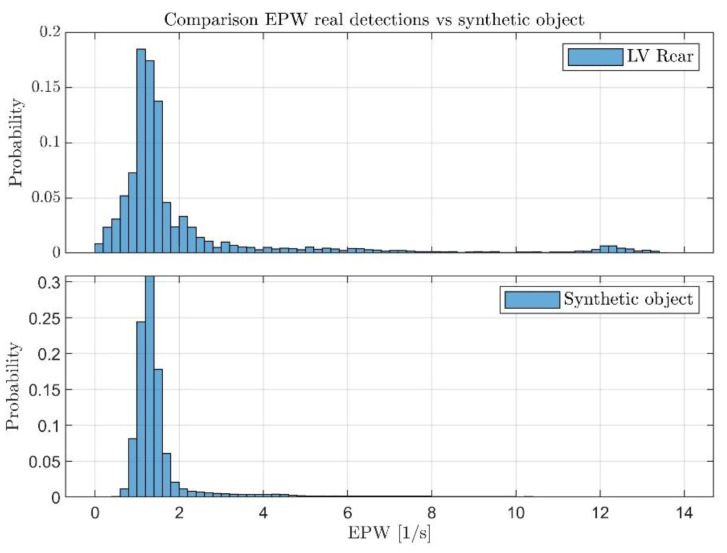
Histograms showing the probability distributions of the EPW values for the rear part of the real LV (up) and the synthetic object (down). The mean and standard deviation as obtained after rendering the scene for the synthetic object are adjusted to resemble those of the LV.

In order to find problematic regions in the parameter space, Figure 9 shows the results obtained by using the previously trained classifier on smaller clusters of the data. Here the focus is on the effect of changes in the obstacle size and the position of the LV. Further dependencies can be seen in the Figure A2. 

Regarding execution times, a single threaded, not optimized version of the proposed algorithm is required to extract the feature vector and complete the inference of 182 ± 51 ms per frame, using C++ in a computer with an Intel CORE i7-8850H CPU. The variation was caused by the changing number of detections. However, current platforms used for AV like RENESAS R-Car and NVIDIA Drive integrate accelerators for image processing tasks [35,36], which, given our use of fixed histogram sizes, can be employed to highly parallelize the computation, reducing the execution time. If for example three consecutive sensor frames are used, this corresponds to ~120 ms [37], and segmentation of the ROI for each of those frames should take few milliseconds, leaving around 150 ms for the calculation of the feature vector and classification which should be attainable. The time required to generate the synthetic data and train the neural network is not included as it is assumed that it can be done offline. The pretrained network can then be used in a vehicle and updated if needed over the air.

## 4. Discussion

Figure 9 shows that the classification becomes difficult when the center of the box moves closer to the road boundary $SO_{y}$ < −2.5 m Figure 9a,b. This is due to the multitude of shapes that the road boundary can generate (see Figure A1), which can be easily confused with a rectangular-shaped object. Additionally, when the LV moves into the ego lane while overtaking $LV_{y}\;$ < 2.25 m Figure 9d, the accuracy of the classifier is reduced as it probably learns that when there is an object in the ego lane, it is most likely an obstacle. Something similar happens when the synthetic object is far away from $SO_{x}$ > 16.5 m Figure 9c. In this case especially, if the box is not big enough and there is lots of spray, it is likely to result in a misclassification. Finally, when the LV is quite far to the left $LV_{y}$ > 4.25 m Figure 9d or close to the beginning of the ROI $LV_{x}$ < 15.5 m Figure 9c, only a portion of the vertical sideline of its shape can be seen. This is easier to misclassify than the usually expected ‘L’ shape. Although these regions are challenging to classify, accuracy in general is high. This would not be the case if the probability of finding objects in the driving lane for the used ROI were not as low. For instance, at lower speeds or in urban traffic the task would be more challenging, as the position of the synthetic object would not be as important a classification parameter as it is on a highway at high speeds. Additionally, although care was taken to make the synthetic data as similar as possible to the real data, it can be that some of the parameters are still different enough to make the classification task easier. For instance, a unique reflection value was used while real obstacles would probably be composed of different materials with different reflection values. 

The presented model constitutes a basis upon which more complex scenarios can be simulated with relatively small changes. For example, the type of synthetic object can be easily changed and its position can be animated or simulated as desired. Their number can also be increased and they do not have to be static. For instance, they can simulate other vehicles. Real and synthetic data can also be combined in different ways. The spray could be generated completely synthetically, keeping just the real detections caused by the LV, or a synthetic LV can be used leaving just the real spray detections. 

The proposed methodology is based on open-source tools and can be easily applied to other LIDAR sensors. It can also be used to simulate other weather-related effects. For instance, the simulation of the detections caused by exhaust gases.

A certain amount of data collection is needed as many of the simulation parameters are extracted from the characteristics of the real point clouds. It is, however, a much easier collection task as no real obstacles need to be present on the spray region.

## 5. Conclusions

Regarding future research directions, even though a shallow neural network was considered enough for the current use case, the use of a more sophisticated network type and architecture can increase classification accuracy. This is especially true if other obstacle types are introduced, for instance bicycles, tires, vehicle parts, etc., in which case manually defining the classifier features is difficult. Additionally, even though only one reflection value for the whole obstacle object was used, a texture can be used to give different parts different reflection values. More complex physics can also be added for the obstacle, such as bouncing or breaking apart.

## Figures and Tables

**Table 1:** Highway measurements (taken from [10]).

Date	Duration	Rainfall
13 April 2018	1 h 17 min	2 mm
16 May 2018	3 h 26 min	3.6 mm
17 May 2018	2 h 20 min	3.1 mm

**Table 2:** Number of particles used.

Particles	Number of Particles	Distribution	Mesh Diameter
Free	~50 per wheel per frame	Defined by acting forces	1.8 cm
Confined	200.000	Uniformly	6 mm

**Table 3:** Classification results.

		Predicted Class			
		All Durations		Duration 3f	
		NB	B	NB	B
Actual class	NB	12,454,236	20,036	4,150,856	6686
	B	3848	141,545	1178	47,055
	F-Score	99.90	92.22	99.91	92.29

## Data Availability

Not applicable.

## Abbreviations

ADAS	Advanced driver assistance systems.
AV	Autonomous vehicles.
EPW	Echo pulse width.
GPS	Global positioning system.
IOR	Index of refraction.
LIDAR	Light detection and ranging.
LV	Leading vehicle.
OI	Object index.
ROI	Region of interest.

## Appendix A

Some of the road boundary shapes are shown in Figure A1a–c. Figure A1d shows another vehicle. As can be noticed, the road boundary can generate shapes that can be easily confused with other objects and, due to curves, it is not limited to a specific region. Other vehicles like trucks can also generate complex point clouds.

## Appendix B

Matrices used in the convolutions: impulse (A1), horizontal (A2) and corner (A3). These matrices are used to identify features such as isolated detections as present in the spray cloud or parts of the synthetic object or leading vehicle.

(A1) $$\left(\begin{array}{ccc} -1 & -1 & -1\\ -1 & 4 & -1\\ -1 & -1 & -1 \end{array}\right)$$

(A2) $$\left(\begin{array}{cccccccccc} -1 & -1 & -1 & -1 & -1 & -1 & -1 & -1 & -1 & -1\\ 2 & 2 & 2 & 2 & 2 & 2 & 2 & 2 & 2 & 2\\ -1 & -1 & -1 & -1 & -1 & -1 & -1 & -1 & -1 & -1 \end{array}\right)$$

(A3) $$\left(\begin{array}{ccccc} -1 & -1 & -1 & -1 & -1\\ -1 & 2 & 2 & 2 & -1\\ -1 & -1 & 2 & 2 & -1\\ -1 & -1 & -1 & 2 & -1\\ -1 & -1 & -1 & 2 & -1 \end{array}\right)$$
